# What does it mean to conduct ethical research after disasters? A case study of the 3.11 disaster in Japan

**DOI:** 10.1111/disa.12681

**Published:** 2025-03-04

**Authors:** Sudeepa Abeysinghe, Kaori Honda, Claire Leppold, Allison Lloyd Williams, Akihiko Ozaki, Aya Goto

**Affiliations:** ^1^ University of Edinburgh United Kingdom; ^2^ Tohoku University Graduate School of Medicine Japan; ^3^ Melbourne School of Population and Global Health Australia; ^4^ Lancaster University United Kingdom; ^5^ Jyoban Hospital of Tokiwa Foundation and Fukushima Medical University Japan; ^6^ Harvard T.H. Chan School of Public Health United States, and Fukushima Medical University Japan

**Keywords:** 3.11, community engagement, ethics, Fukushima, interviews

## Abstract

The 3.11 disaster in Fukushima Prefecture, Japan, is a useful case study through which to interrogate research ethics. This region has been the site of a high degree of research interest, which sometimes presented a source of stress to local communities. This study examines researcher perspectives on the ethics of post‐disaster health research. Qualitative interviews were conducted with these informants, who noted that recovering communities experienced significant over‐research, particularly in the form of survey fatigue, which was seen to influence viewpoints concerning both recovery and agency. Efforts to integrate better into community needs reoriented reflexive research towards ‘post‐normal’ forms of working. Simultaneously, researchers had to navigate funding and reward structures that prioritised the swift production of results. Focusing on community engagement and feedback, and managing this ethical complexity, were seen as essential forms of ethical practice to mitigate the negative impacts that the influx of research activity can have on a recovering community.

## 1 INTRODUCTION

The ethical impact of research on disaster‐affected communities is a topic that has been garnering increased attention (Van Brown, 2020; Schobert et al., 2023; Adams, Evans, and Peek, 2024). The status of these communities as specifically vulnerable and in need of special ethical measures has been a core subject of deliberation. Such concerns are particularly pronounced in relation to the conduct of health and medical research within disaster settings. Here, personal and sensitive information can constitute essential data, collected in a context in which communities have experienced challenges to their health and well‐being as an outcome of disaster (Mezinska et al., 2016). Debates within the published literature on the ethics of health and disaster research focus on whether such work presents unique ethical questions within an institutional ethics review process (for a scoping review, see Abeysinghe and Leppold, 2023). These questions tend to relate to ethical areas of risk and vulnerability (Schobert et al., 2023), and have concentrated in particular on the perceived increased vulnerability of research participants as a result of post‐traumatic stress (O'Mathúna, 2010; Tansey et al., 2017).

A smaller, but illuminating, subset of the academic literature centres on the practical ethics of health and disaster research. These works take the form of reflexive accounts of researchers concerning the challenges and contexts they encountered during discrete research projects. For example, some researchers focus on participatory or action research as a more ethical approach to studying community needs post disaster (Pyles, 2015; Lesen et al., 2019), and others note that heightened reflexivity is required when conducting research in disaster settings (cf. Allden et al., 2009; Chiumento et al., 2017; Kita, 2017). Researcher reflections on practical ethics are an important resource in developing best practices, highlighting areas where further guidance or attention is needed, and moving the debate on disaster research ethics beyond a spotlight on the formal ethics review process and towards interest in the impact of research as a social practice. Advancing beyond individual accounts to the collective experiences of researchers is necessary to place a broader lens on this issue, drawing on common threads of reflection and activity across researchers who possess experiences under similar field conditions.

The 3.11 earthquake, tsunami, and nuclear ‘triple disaster’ in Japan is an instructive case via which to analyse collective researcher experiences of ethics and research practice. The time that has passed since the disaster (11–12 years at the time of our data collection) has allowed space for reflection, and the wide variety of short‐term and ongoing health research methods and practices surrounding the disaster mean that commonalities across research types can be drawn out. The impacts of 3.11 are far‐reaching and long‐lasting within Fukushima Prefecture and the wider Tōhoku region. Following the disaster, there was a sharp increase in research activity, mirroring a common experience of other disaster‐affected areas (Patel et al., 2020; Wordsworth et al., 2021; Ingham et al., 2022). This work involved academics and practitioners who had previously been connected to the region, as well as those drawn to the area as a site of research following the disaster. For example, Yamamoto et al. (2015) conducted a survey of health and medical research, citing 90 such studies. While the peak of research activity occurred during the years immediately after 3.11, there is still an ongoing researcher presence in the Tōhoku region. The ethical impact of this cumulative research presence is significant in terms of interrogating the wider benefits and harms caused by research to these communities, as well as the ethical reflections that have been published by researchers in the time since the disaster.

Post‐disaster health research in Fukushima Prefecture and the wider Tōhoku region has produced some notable controversies, with citizen groups, medical professionals, and other academics contesting the research processes, results, and public health implications of projects or programmes of work (Kobashi et al., 2020; Sawano et al., 2023). The health impacts of radiation have been a key site of contestation (Yasui, 2013; Polleri, 2020), and this has been associated with the rise of citizen science (Kimura, 2019; Polleri, 2019; Koyama, 2022), which has become a routinised form of developing and asserting lay expertise. The broader socio‐historical context of the disaster, and post‐disaster research practice in general, also frames the ethical landscape. Miyazawa (2018), for instance, notes that distrust of biomedical researchers following 3.11 is linked to the historical memory of ethics violations experienced by communities after the atomic bombings of Hiroshima and Nagasaki in 1945. As is common across other disaster settings (Refsdal, 2014), the region also received a high number of ‘parachute’ researchers, conducting one‐off studies or longitudinal forms of data collection at distance, which is often experienced as unsettling or exploitative (Elliott, 2013; Matsumoto, 2013). These histories frame the broader engagement between the local communities and researchers, both as individuals and as collectives.

Health research in disaster contexts, as exemplified in the case study of the 3.11 disaster, necessarily engages with these existing parameters of community knowledge and experience. Within the field of Science and Technology Studies (STS), this form of research activity is referred to as ‘post‐normal science’ (Funtowicz and Ravetz, 1993; Ravetz, 2004), where the conditions under which research is undertaken have shifted from an emphasis on the interests of scientific disciplines (taken broadly, to include all academic fields) towards negotiating and interrogating the questions posed by community, societal, and policy needs. Within the STS literature, this idea is most commonly applied to the area of environmental research (Saloranta, 2001; Howard, 2011), where programmes are led by scientists but respond to real‐world needs, rather than purely the types of questions that would be derived from siloed disciplines. This is known as ‘strongly contextual’ or ‘highly integrated’ research (also referred to as ‘mode‐2 science’), where the focus, form, and outputs of research speak to the contexts in which research is undertaken (Nowotny, Scott, and Gibbons, 2001). This frame to examine research activity is applicable to 3.11. The rise of citizen science and citizen advocacy groups, the need to respond to uncertainties around radiation exposure, and the politicisation of both the disaster response and the scientific knowledge base all underpin the ways in which research is experienced and conducted in this setting.

This mode of research is of specific importance in the 3.11 case, and understanding the ethical implications of strongly contextualised research is also an issue of wider interest. For example, ideas of co‐production are now widely adopted in policy spheres (Wyborn et al., 2019; Galende‐Sánchez and Sorman, 2021) and increasingly integrated into research funder expectations, in the form of public or policy involvement in research design and methodology (Bovaird et al., 2015; Howlett, Kekez, and Poocharoen, 2017; Smith et al., 2022). These forms of co‐production go beyond initial STS usage of the term to situate community‐engaged research practices more widely as valuable in counteracting power asymmetries and producing more socially‐useful forms of knowledge (Olesen and Nordentoft, 2013; Filipe, Renedo, and Marston, 2017; Lambert and Carr, 2018; Thomas‐Hughes, 2018). Understanding the ethical consequences of this mode of research is therefore of more general relevance, and the post‐disaster Tōhoku region offers a pertinent setting within which to examine this question.

## 2 METHODS

This study assesses research narratives of ethical practice in the post‐disaster context of the Tōhoku region following 3.11 through semi‐structured key informant interviews. It poses the following question: how do health and disaster researchers understand ethical practice, the limitations of current research practice, and the potential for improvement, based on their experiences of the 3.11 disaster?

Sampling focused on recruiting researchers with diverse experiences. The initial sampling strategy entailed a scoping of the published literature through a PubMed search to identify authors who had written on health and 3.11, using a combination of the search terms of Fukushima and Japanese equivalents (such as 福島), as well as descriptors of the disaster (earthquake, tsunami, nuclear) and the field of research (health, medicine). The results were cross‐referenced with researcher webpages, noting Japan‐based researchers who had been active in the field (rather than, for example, being among the authors of a single paper). This generated a list of 44 researchers. Further exclusion of researchers whose main focus was not health reduced the number to 30. Recruitment was initiated with this list of 30 potential interviewees. Following this, participants were generated through the research and professional networks of authors KH and AG and snowball sampling.

The overarching sampling strategy privileged a diversity of participants in relation to several characteristics: disciplinary background (studying health across the humanities and medical, natural, and social sciences); research methods (spanning different quantitative and qualitative research methods, including surveys, ethnographies, sample collection, and medical testing); career stage; and varied professional backgrounds, including full‐time academics as well as those conducting research but who primarily identify as practitioners. The study concentrated on Japan‐based researchers, encompassing both those who saw themselves as ‘local’ to the field and those from beyond the Tōhoku region, with the final sample incorporating researchers from across Japan. The study yielded a final pool of 14 participants, and the core themes of the data reached saturation. Table 1 shows the characteristics of the key informants interviewed.

**TABLE 1:** Aggregated profiles of the key informants.

Characteristic	Participants
*Gender*	
Male	9
Female	5
*Discipline*	
STEM (science, technology, engineering, mathematics)	Including: medicine (2); nursing (2); clinical psychology (2); midwifery (1); health technician (1); engineering (1)
SHAPE (social science, humanities, arts, people, economy)	Including: anthropology (1); sociology (1); social psychology (1); legal studies (1)
*Methods* *	
Quantitative and laboratory‐based	Including: quantitative (8); physical field studies (2) experimental (1); sample analysis (1)
Qualitative	Including: qualitative (6); ethnography (1)
*Residence*	
In Fukushima Prefecture at the time of 3.11	4
Not in Fukushima Prefecture at the time of 3.11	10
*Career stage*	
Self‐description of current career stage	Including: professor (5), lecturer (3), specially‐appointed professor (2), assistant professor (2), principal investigator (1), early career researcher (1)

**Notes:** Disciplines and methods are described broadly to avoid deanonymisation.

* The number of listed methods exceeds the number of interviewees owing to multi‐method researchers.

**Source:** authors.

Following explanation of the interview process and the collection of informed consent, each semi‐structured interview investigated a number of key topics through an interview schedule developed by KH, SA, and AG. The interviews probed the following areas: experience of the formal ethics review process; ethical issues and conditions confronted while in the field/undertaking data collection; interviewee suggestions about good ethical practice; challenges to good ethical practice; and a set of broad questions to understand each key informant's own ethical positioning and outlook. While these areas were explored with each participant, the interviews followed a semi‐structured form that adapted to the context of each interview encounter. The interviews were conducted by KH between December 2022 and May 2023. Owing to the COVID‐19 (coronavirus disease 2019) pandemic in Japan, most of the interviews were conducted online; two local interviews were held face‐to‐face. The length of the interviews ranged from 58 to 94 minutes, averaging 75 minutes. Interviews were transcribed and checked in batches.

Prior to formal analysis, the data were anonymised and translated by KH. After translation, they were checked for accuracy by KH and AG, and then underwent pilot coding using a thematic analysis technique (Braun and Clarke, 2006). Thematic analysis was deemed the most appropriate approach given the exploratory research question and the translated form of the data. Initial pilot coding was undertaken, independently, by SA and KH, who then met to discuss the approach. At this point, general thematic findings were also presented at a dissemination event held in Fukushima Prefecture (July 2023) to cross‐check with interviewee perspectives. Following these forms of inter‐coder validation and participant validation, the coding strategy was finalised, and the English transcripts of the interviews were fully coded by SA.

In conducting this study, we attempted to maintain a reflexive stance in terms of our own embeddedness in the field. The research team is composed of individuals from across career stages and disciplines (epidemiology, medicine, nursing, public health, social science). All members have themselves conducted research on 3.11, together and with other research teams, variously from local (AG, AO, KH), insider/outsider (CL), and outsider (SA, ALW) standpoints. In addition to our own experiences in the field, the core research question was a result of informal discussions among researchers about the ethical complexities of this post‐3.11 work. In relation to procedural ethics, a favourable ethical opinion was provided by the Research Ethics Committee of the School of Social and Political Science, University of Edinburgh, Scotland (ID 284739).

## 3 RESULTS

The interviewees emphasised the problem of over‐research among the local population, exacerbating the stress following the 3.11 disaster. As a result, they reported that they attuned their understandings of ethical practice and thought more deeply about the structures of research that underpinned this community stress. Ethical action included attempts to refocus their research activities and engage more profoundly with the communities, demonstrating a move towards more strongly integrated (‘mode‐2’) research.

### 3.1 Research fatigue and participant stress

A principal finding of the interviews was that post‐disaster studies had caused research fatigue and stress among people who had experienced the 3.11 disaster. Interviewees reported that this stemmed from the profusion of surveys that were distributed to citizens, a phenomenon that has been anecdotally noted by others and was reflected on in great depth by the key informants.^1^ Research fatigue was seen by the interviewees as a driving ethical impact of disaster research. The interviewees recounted that the problem of ‘survey pollution’ was well‐known by researchers and community members in respect of 3.11:

*This is something that everyone said, not just me personally. Especially in the years after the earthquake, not only the survey forms from [a university], but also from the municipalities, the national government, general companies, and anyway various people have been sending more and more mail to the victims and the mailboxes have been full. Or people who had evacuated and were living [somewhere else] without saying they were evacuees would also be revealed if they had envelopes in their house. I've heard many stories like that, so it wasn't so much my own personal sense of the problems, but rather that everyone knew what they were doing, but we had to do it* (STEM researcher 6).

In addition to describing the problem of research fatigue, this quote reflects some of the tensions in conducting research in this context; many of the interviewed researchers saw it as imperative to collect their own data, even though they were simultaneously aware that residents were burdened by surveys. As this interviewee suggests, many researchers felt that they ‘had to do it’ in order to gather, preserve, and intervene in the strengthening of the health outcomes for the region.

Several interviewees characterised this over‐research in more uniformly and forcefully negative terms. For example, one participant ascribes this form of research fatigue to parachute researchers:

*At that time, people often said things like [they were] being treated as guinea pigs. Well, I heard that a lot. I joined the project [at a particular career point], so I was in it after a lot of people had left, like a swarm of ants. So, from the residents' point of view, it's as if I came after they had been overrun for a long time… I have not met those ants. I'm in there after the ants are already gone. So, I think they had the impression that the people who came in like those ants, they just took what they wanted to take and left quickly. In that sense, I think it's about at least sustaining the relationship, or at least respecting the target group, the other person, and their human rights. I think that's all there is to it* (STEM researcher 6).

As well as constructing a distinction between short‐term researchers and those in the field after the acute phase or engaged in long‐term research, this quote also speaks to the value of embeddedness in the community. This was commonly regarded by the interviewees as a key ethical virtue and is appraised in further detail below.

In understanding research fatigue, the interviewees emphasised that the disaster context itself exacerbated the negative effects of over‐research, especially concerning the mental and physical health surveys conducted following the disaster. The informants felt that the over‐saturation of such projects had the potential to reinforce and aggravate the psychological impacts of 3.11, by placing a focus on the disaster and continuing to label people in relation to their experience of it. One pointed out:

*I think that the survey as a survivor makes you fix your position, or forces you to think about it. We have talked about how they are made to remember things they don't want to remember, and also how the survey can instil a negative attitude, like ‘I am a survivor after all’* (STEM researcher 2).

The interviewees reflected that experience of over‐research was magnified in the wake of the disaster. Questions about psychological well‐being prompted continuous probing, across multiple years, of this sensitive point:

*In the first place, you are in a situation where you are in a lot of trouble yourself. Even in normal life, it's a hassle to answer questionnaires, isn't it? And yet, I received a questionnaire at such a difficult time. Moreover, there are many questionnaires that I don't even know how they differ …we were asked over and over again, ‘Are you happy?’. You don't usually think about whether you are happy or not, but they keep asking you to answer that question …and it's like, ‘I answered yesterday’, or ‘I answered earlier’* (SHAPE researcher 1).

These quotes are examples of the importance of research fatigue as an ethical issue, and this was a concern that was articulated repeatedly in our interviews, with many participants going into great depth on this topic. This issue therefore forms a prism through which much of the discussion about research and researcher ethics was viewed by participating researchers in relation to 3.11.

One of the aspects of research fatigue that is potentially specific to 3.11, and which informs the depth of informants' reflections in this area, is the circumstance that many of the interviewed researchers were locals to the region. This facilitated the experience of research fatigue from the dual perspectives of an outsider (researcher) and, in some cases, also an insider (potential participant). In the words of one interviewee:

*I was provided with temporary housing in the village, and I lived there, and I received an awful lot of survey forms. When I opened the post box, I found that there were as many survey forms as this. I am not a villager, so I cannot answer them, but it was an opportunity for me to experience what the villagers must be going through. The village was really like a test subject, and I received a lot of surveys several times a month, and I experienced what it is probably like to be the victim of the so‐called survey* (STEM researcher 3).

This idea of being a ‘victim’ of research, following the experience of surviving and rebuilding after a disaster, is articulated throughout the interview data. This reflection also speaks to the notion of research victimisation through continued negative experiences of research (such as repeated surveys over many years), which was prolonged in the service of the researchers or organisations conducting the survey‐related work.

Researchers were aware at the time of overuse of survey instruments being an outcome of the activities of the research community as a whole. There was strongly expressed sympathy regarding the impact on local community members. Simultaneously, many of the interviewed researchers also needed to rely on survey instruments to conduct their own studies. One said:

*I gradually learned that I had a bad experience with a lot of questionnaires, as I mentioned earlier, only after I got into [the field site] and heard about it… So, I did not just conduct the survey out of the blue, but rather, I conducted the survey while learning about the situation of the people being surveyed and the people who were cooperating with me. I think it was good that we were able to conduct the survey while learning about the situation of the people we were surveying and the people who were cooperating with us* (SHAPE researcher 1).

This position, of having experienced the impact of survey fatigue but also contributing to the proliferation of surveys in the community, leads to an attitude of reflexivity on the part of the researchers interviewed. As one put it:

*On the other hand, from the viewpoint of subject protection, of course, if you were asked on paper once every two months whether you feel like dying or committing suicide, you would get fed up. We have to protect those points …it's difficult. I thought it was very difficult to find the right balance in the field* (SHAPE researcher 3).

In this manner, individual researchers found it hard to weigh their own professional purpose against their understanding of the wider footprint of the researcher community in the field. This tension was emphasised in their experiences of the structural drivers of research.

### 3.2 Structural constraints of the research environment

In locating the impacts and implications of researchers on a disaster‐affected community, the role of the wider structural context in which 3.11 researchers found themselves presented itself as another important site of ethical reflection. While this thread was less universally noted across our informants, several researchers suggested that the structural characteristics of research work are a key determinant of the highlighted ethical concerns. For example, one researcher identified the issue as pertaining to the funding systems:

*[I]t creates a structure in which various research funds are spread around, so the structure is such that research funds are taken immediately, but once they are taken, a questionnaire has to be completed. There are a lot of researchers who are in need of research funds, so they have to take various surveys from various regions, and this social structure produces survey pollution* (SHAPE researcher 3).

Another informant noted that the prevalence of parachute researchers in disaster sites is at least partially a result of the funding apparatus that they are working within, but underlined that such constraints should not negate ethical practice:

*I think that it is also true that [researchers] disappear suddenly. I don't think it's right that they say that they will leave because the budget has run out. From a long‐term perspective, I think it is important that both parties agree on the limits of the project* (STEM researcher 2).

The short‐term nature of research funding is seen here as a key contributor to the outcome of research fatigue.

Other systemic aspects of the research process were perceived as contributing to the negative experience of communities. In relation to over‐surveying, the mechanisms through which surveys are distributed were also flagged as a contributory factor:

*There are a lot of companies that make money by sending out these envelopes from different places every week, so the money goes to them and the questionnaires are constantly sent out, and that's the way it's structured. It is important to say that it is better to prevent this from happening …but I don't think that answers the question of how we are going to eat then [that is, how will researchers make a living]* (SHAPE researcher 3).

This suggestion implies that good ethical practice, at the level of the research community as a whole, requires more fundamental reflection on the sometimes taken‐for‐granted mechanics of research design and the systemic contradictions of academic practice.

Another structural issue lies in the perceived lack of coordination among the research community. The atomistic nature of the research enterprise is highlighted in the following quote as contributing to the issue of research fatigue:

*I think that each of us is conducting the research from our own standpoint, but if you think about the subject or the people, I think that, to some extent, the researchers should be united if they are going to conduct the same research, and I think that such a perspective would be very lacking. I think that this perspective was very much lacking* (STEM researcher 2).

Further efforts at coordination around survey instruments is suggested as a potential best ethical practice in these contexts, given that duplication of materials is a characteristic of the proliferation of surveys. This might be challenging, however, as one interviewee emphasised:

*This issue of duplication is difficult. It's a question of who checks it. There is no such thing as overlapping studies, so it's just a matter of asking similar questions but from slightly different perspectives… If it were me who was to decide on this, I would say, ‘Why is this [one survey] not good?’ [as opposed to another]. This is quite a difficult issue* (STEM researcher 4).

The potential to coordinate research better within disaster settings is a key ethical insight. Yet, structural aspects of the research ecosystem—such as funding and the incentives of career progression—would seem to undermine the likelihood of some forms of coordination arising as applied ethical practice in these contexts.

### 3.3 Trust and community integration as an ethical practice and a research skill

Drawing away from the structural focus, at the level of individual research practice, the interviewees reflected at length on the importance of community trust. The 3.11 case therefore demonstrates the prominence of strongly contextualised research, in part as a product of ethical complexities resulting from highly researched populations.

Connected to, but broader than, the issue of research fatigue, from the perspective of the interviewees, is that the ambiguity of the research process led to a lack of community trust. One interviewee, explaining his simultaneous position as a researcher and presumed participant, pointed out:

*[M]oreover, they send them [surveys] to me out of the blue without my permission, and it is common for me to just send them to them and never hear from them after I have answered them. It's like it's only natural, because people won't cooperate with that* (SHAPE researcher 1).

Despite the general approach of survey researchers using third‐party companies for survey distribution, in relation to 3.11, the informants noted the importance of in‐person engagement. While such methods practices are common for practitioners of qualitative research, they were also adopted as ethical practice by quantitative and natural scientific researchers in this setting. One interviewee stated:

*I think that a faceless relationship, such as a telephone call, or a document exchange, or a complete e‐mail exchange, is very likely to give the other person a sense of distrust. I think it is important to have a face‐to‐face relationship, and to have time to introduce myself and the researcher to the target audience, to explain to them who I am and what I do, and also for the target audience to explain to the researcher who they are. The subject also takes time to explain to the researcher what kind of person they are* (STEM researcher 1).

In this manner, the research process itself is seen as an act of exchange, curbing some of the potentially extractive elements of research activity. In‐person engagement and, ideally, sustained contact with the local community were seen as essential to ethical practice by those interviewed. Even long‐term research was queried in cases where it was not accompanied by a meaningful community presence:

*I think that basically anyone who cannot go to the site once a week should not do it. I don't know …but [a university in western Japan] has said in about five years that it has been to [a site in] Fukushima three times so far. I think it would be a good idea to stop all such things, such as conducting mental health surveys and saying ‘we have been there three times before’. In reality, I think the best way to get in is to say, ‘If you can't go at least two or three times a month, then don't do it’* (SHAPE researcher 1).

It was understood, then, that the process of collecting data, not only the outcomes of research, needed to be concerned with the well‐being of the community for the research to be ethical. There is a practical element here, too, as interviewees acknowledged that obtaining data in this context is difficult without building trust with the local community.

Many of the participants spoke about trust as formed through engaging with gatekeepers and community leaders, a strategy that is ‘significant in terms of personal connections, whether or not we have that’ (STEM researcher 4). For outsider researchers, such relationships are invaluable as it ‘is impossible to suddenly come in and build trust’ (STEM researcher 6). Such strategies can be seen as supporting ethical survey practice, even with respect to this over‐surveyed population. In the words of one interviewee:

*[W]e need to be grateful and respectful of their cooperation, and also, in terms of research, they are more likely to respond if we do so. It is important to get them to cooperate, and in the long run, they will not accept the survey because they have been repeatedly responding to a cursory survey. If they say they won't accept the research, then researchers can no longer do the research there. They can no longer do research. If we can no longer do research, we will have no way of knowing if these people are in fact at risk… [I]t will be impossible to collect data after that, creating a negative vicious circle* (SHAPE researcher 1).

As the quote above suggests, such strategies are pragmatic, since good data cannot be collected without these forms of integration.

Such engagements, though, were not solely strategic, but were also viewed as part of the ethical attitude towards research. Engagement with the community as a matter of practice was seen to ensure that the needs of the community were centred within the research (‘mode‐2’ research), rather than the research being centred on the abstract questions of the researcher (‘normal science’). The ethical positioning of the researcher was perceived as one that works with, and within, the community of participants:

*I think it is necessary to have an attitude of thinking together with local people. If you go in with a fixed idea, you will end up with conflicts of opinion, or you will end up forcing your own ideas on them or trying to persuade them* (STEM researcher 4).

Simultaneously, forms of value neutrality were regarded as potentially undermining ethical practice. In respect to 3.11, contestations regarding the health impacts of the radiation aspect of the disaster were often borne out through formal research studies. Consequently, it is necessary for the researcher to make their own presuppositions and hypotheses clear to potential participants, reflexively acknowledging that the former's reading of research data is likely to be constructed in relation to pre‐existing orientations. As one interviewee stated:

*[A]lthough it sounds very contradictory, if you say that you come from a neutral position or that you are here purely for the people, you will not be trusted… I think that when people say that they are neutral, but in a context where the situation is clearly polarised, they don't trust you if you say that you are totally neutral or that you will [impartially] listen to what they have to say. It is better to say that I am a person in this position, that I want to do this kind of thing, and that I came here because I wanted to hear this kind of thing* (STEM researcher 6).

As with deeper community embedding of survey and other quantitative researchers in this context, experience of the needs of the local population also nudged researchers towards forms of reflexivity more commonly aligned with qualitative approaches. Here, they discard ‘value neutrality’ for an explicitly articulated position regarding the research objectives. Ethical research practice in this situation thereby oriented itself towards the community and required forms of community embeddedness that might not be seen as a requirement by quantitative research traditions in other settings. This form of reflexivity is also extended to the post‐research practices of the interviewees.

### 3.4 Post data collection: dissemination and leaving the field

In addition to the problem of research fatigue and over‐research, another overwhelmingly recurring theme of the interviews concerned the feedback of results to participants. This process was understood as having not (always) been conducted well by 3.11 researchers and was seen as a crucial area for improvement.

The interviewees suggested that feedback to participants is a particular ethical skill that needs to be more routinely incorporated in researcher practice. One remarked:

*When I look at the various studies that are being developed here, there are people who are really good at giving feedback. I think it would be very different if everyone did it this way… Research presentations at universities tend to focus on the main contents of research. But I think it would be good to exchange a bit of information on how to give feedback* (STEM researcher 1).

The research community was seen as currently needing further training in the skill of communicating feedback to participants. For example, many interviewees commented that some researchers, while commendably attempting participant feedback, ended up delivering it in unhelpful ways. One said:

*To be honest, I think that the results are still being explained in terms that are difficult for the general public to understand, even though people like me might understand them if we look at them from a specialist's point of view. So, I honestly think that there should be more ingenuity in the way the research results are returned, yes* (STEM researcher 10).

Providing feedback that was both mindful of the sensitivities in the data but presented in a digestible manner was understood by the informants as a vital, and potentially rare, skill among health and disaster researchers following 3.11. For instance:

*Of course, it's not about forcing them to ask, ‘is this what the psychological damage is like?’. It's about giving them information in a way that is easy to understand, at least for the people of Fukushima… It is not the end of the press release or the research paper at the university, but at least a polite and detailed statement to the people in the area who have been helped, or who have been surveyed* (STEM researcher 9).

This quote reflects some of the unease around this aspect of the process. Forms of public feedback were seen as ethical practice but were also experienced by researchers as discomforting, perhaps particularly because of the highly integrated nature of 3.11 research. For example, in the context of a politicised post‐disaster environment, researchers found it both necessary and difficult to present results that might be contradictory, hard to grasp, or may not offer a clear explanation of the situation. One interviewee observed:

*[E]ven if a researcher feels that presenting his or her research results might cause confusion, I don't think it is necessary to refrain from doing so. However, I think it is necessary to think carefully about how it will be communicated and what effect it will have* (STEM researcher 10).

In addition to confusing results, the findings of researchers' studies were sometimes at odds with the expectations of community members:

*[W]hen they have already decided what they want to hear, nothing you say will resonate. It is only when you say exactly what they want to hear that you get a reaction. In fact …when you report a bit more honestly, for example …that you don't know what will happen to Fukushima in 10 years' time, you are no longer an expert [in the eyes of the community members] if you don't know. Some people responded that if you are an expert, you should answer one way or the other, ‘yes’ or ‘no’… In the end, there is a dilemma as to whether researchers should respond to the voices of residents who want a clear statement… It is sincere to say that we didn't understand, or that we still don't understand, but it can also stir up dissatisfaction among the residents on the other side… Yes, there's always the feeling that you won't be able to meet all the expectations* (STEM researcher 5).

One potential ethical complication of highly integrated research may be the possibility of greater dissatisfaction when the findings either do not match community expectations or where the messiness of the findings means that they do not satisfy the expectation that the research will resolve the uncertainties experienced by the community.

A related complication in terms of embeddedness is the matter of how a researcher can ethically extract themselves from the field. While this was a less frequently noted aspect among the informants, it still warrants some illustration. For instance:

*When to leave the involvement. I myself had a rather difficult time with this, or rather, I was troubled by it. After all, if you are involved from the outside, you are not a person who stays there forever, are you? …I don't think that relying on [outsider/researcher] support all the time is the way it should be. So, I think it was difficult to decide when to stop involvement* (STEM researcher 2).

This indicates another area of complexity for disaster researchers engaged in highly integrated modes of research practice. On the whole, such forms of integration were seen by our interviewees as pivotal to ethical practice in the 3.11 context. Ensuring that research is embedded in the concerns of the citizens, and that findings are appropriately fed back to the community, are perceived as vital ethical practices. Nonetheless, such forms of research and engagement highlight tensions that require further investigation, as well as pinpointing areas where the researcher community can be further supported in its practice.

## 4 DISCUSSION

Following the 3.11 disaster, health researchers found themselves exposed to the limitations of some disciplinarily dominant research strategies that privilege ideas of objectivity and detached data collection. The proliferation of survey research, in particular, prompted ethically reflexive researchers to negotiate their own practices in a more nuanced way. This led to reorientations of research towards modes of work that prioritised more integrated strategies, as articulated by ‘mode‐2’ accounts of research, and to an increased understanding of the experiences of research participants.

Research fatigue is a frequent experience of disaster‐affected populations, as noted in the wider disaster studies literature (Brun, 2009; Refsdal, 2014; Patel et al., 2020). Disaster contexts attract a variety of researchers, and while each may be individually well‐meaning in their intention to learn from the disaster to improve future outcomes, the 3.11 case demonstrates that the cumulative effects of research can be significant. Extant scholarly literature on disaster ethics tends to focus on post‐disaster psychological distress among potential participants as a form of vulnerability that may impact data collection (Collogan et al., 2004; Levine, 2004). Our informants also reflected on the potential for constant interrogation by researchers to produce a distinct experience of fatigue, including a re‐emphasis on remaining in a state of recovery among community members in post‐disaster Tōhoku (that is, reinforcing the status of people as ‘survivors’ or ‘victims’, in the terms of the informants). There has been limited research on the experience of the vulnerability that might be generated by research activity, rather than the disaster and its management. Yet, there has been some examination of the potential mental health effects of participating in post‐disaster research. For example, in Australia, Gibbs et al. (2018) found that research participants experienced satisfaction from taking part in a study following bushfires, even though at times they experienced strong emotions during data collection; however, these observations were in the context of a single long‐term study that did not take place in the circumstances of high research activity that followed 3.11. Our findings suggest a need to pay particular care to ethics in post‐disaster settings where a large number of studies are occurring, or are about to occur, and when techniques such as repetitive surveys are being conducted by different researchers or organisations without effective coordination. While further investigation of this is needed, it is possible that over‐research itself might have the distinct effect of exacerbating or producing new forms of fatigue and distress. The experience of over‐research can also to some extent limit the ability to conduct research effectively, when community dissatisfaction results in the rejection of further studies. Consequently, the need for researchers to engage in co‐production with the community was presented by our informants as not only good ethical practice, but also to some degree as necessary to collect data within this context.

Our study focuses on an informant pool of Japanese researchers who carried out studies after the 3.11 disaster. This meant that the perspectives of international researchers who engaged in research after 3.11 were not covered here. The greater likelihood of such researchers engaging in parachute research, as demonstrated in other disaster settings (cf. O'Mathúna, 2010; Louis‐Charles et al., 2020), broadens the need to reflect on researcher impacts on local communities that have experienced a disaster. Further investigations concentrating on the collective effect of the influx of international research, together with (or in some cases potentially an absence of) similar levels of domestic research, within these post‐disaster community contexts would be valuable. This might be particularly the case when considering the increased potential for mismatches with cultural expectations and the fact that such effects might be more pronounced in circumstances such as low‐resource settings.

The structural influences on the conduct of post‐disaster research also deserve further attention. Quick‐response research is one of the cornerstones of the approach to disaster studies, and frequently is characterised by the perceived need to collect data proximal to the acute disaster event (Adams, Evans, and Peek, 2024). This is often combined with the proliferation of short‐term funding opportunities (Oulahen, Vogel, and Gouett‐Hanna, 2020). The unintended consequences of these structural influences were emphasised in the interviewees' understandings of the impressions made by the research community on disaster‐affected populations. One significant finding of this study is that the impact of researchers needs to be viewed as a collective. That is, the ethical implications of disaster and health research are experienced by the local community in the form of the cumulative effects of researchers, rather than the ethical implications of single projects (although isolated initiatives may also of course be consequential, especially in cases of ethical mismanagement). The need to examine the collective effects of researchers may indicate a further need to consider the ways that current ethics systems tend to view and assess projects as single entities and the ways that researchers tend to report impact metrics.

The concern about research fatigue raised in the present study demonstrates the cumulative ethical impact of researcher involvement in the region after 3.11. Much of the extant literature on health and disaster research ethics tends to focus either on ethical principles in the procedural ethics system (such as the role of institutional review boards) or take a case study approach vis‐à‐vis the reflexive position within individual research projects or across an individual's career (cf. Abeysinghe and Leppold, 2023). Our informants problematised this spotlight on individual practice and pointed to the need to look at structural drivers, such as common research strategies (including the deployment of external survey companies) and incentives (such as the funding systems in which researchers work), as underlying causes of the poor consequences of research with local people. There are moves within the field to address some parts of this problem. For example, in specific relation to research fatigue, there are calls for research studies to be centralised in future disasters, for instance, by passing all projects through a university local to the disaster site or through a government entity (Gaillard and Peek, 2019). Indeed, following 3.11, the Science Council of Japan (2013) recommended that academic societies prepare a system for identifying and publicising research conducted on the disaster to avoid duplication, although the extent to which this was successful remains unclear. Globally, there are various positive cases of practice that can be drawn upon, such as those involving coordination via ethics review processes (Refsdal, 2014; Adams‐Hutcheson, 2017). Yet, such measures do not mitigate other structural impediments, such as the funding and incentive motivators of researchers.

With regard to 3.11, the interviewees all underlined how a more strongly integrated form of research practice was the outcome of working within the context. Engagement with and integration into community needs were seen as key pillars of ethical practice. The reorientation of some forms of research that would be more susceptible to parachute practices (here, particularly survey research) led, according to the informants, to better observation of the needs and expectations of the community. This was not without difficulty, however, as the uncertainties the informants confronted about aspects of participant feedback, questions of leaving the field and extraction from the research sites, and issues pertaining to negotiating what is needed in terms of good practice in the face of short‐term funding structures, came to the forefront. The interviewees in our study also suggested that further researcher training in working with, and dissemination of research among, lay communities might be required to increase levels of familiarity and comfort with these ways of operating.

These findings should also be viewed in relation to our sample of informants. The interviewees were all researchers, and the data therefore focus on researcher perspectives. The decision to concentrate on researchers was partly in recognition of existing levels of research fatigue in this community and partly due to the importance of learning more broadly from ethical reflections of this type. Additionally, some participating researchers were also living in Fukushima Prefecture and have reflected directly on their experiences as a local resident (and target of other people's research). Furthermore, in assessing the findings of this study, it should be noted that the interviewed informants were researchers who were particularly sensitive to ethics questions, as it was precisely such individuals who expressed interest in taking part in this project when approached for recruitment. Also, many of the interviewees were (strongly or more passingly) connected through networks to members of the research team, owing to the latter also having conducted research following 3.11. Apart from a notable openness among the informants, it seems probable that the interviewees were less likely to criticise other researchers or label specific research projects as ethically problematic, apart from those that have already been widely contested in the media or scholarly literature. The present study therefore recognises that the results do not explicitly constitute findings that are generalisable across all 3.11 health researchers. Rather, the accounts here are synthesised across key informants with a particularly high degree of reflexivity in relation to ethical practice; these are seen to have produced particularly useful insights, spotlighting areas of good and improving practice rather than the poor practice of individual studies.

Understanding the strengths of and the challenges posed by research within a strongly contextualised post‐disaster setting such as 3.11 can strengthen future research practices. Interrogating the activities of researchers as a collective community (in terms of its impacts), albeit a diverse one (in terms of its approaches), is an important lens to continue applying to understand disaster research ethics, going beyond the individual approach that currently dominates the focus of researcher ethics systems and analysing more holistically our shared impact on the communities that have experienced disaster.

## FUNDING

This research was funded by a Great Britain Sasakawa Foundation Butterfield Awards grant (no. B142).

## CRedIT STATEMENT

Sudeepa Abeysinghe: conceptualisation, formal analysis, funding acquisition, methodology, project administration, supervision, writing (original draft).

Kaori Honda: investigation, formal analysis, methodology, writing (review and editing).

Claire Leppold: writing (review and editing).

Allison Lloyd Williams: writing (review and editing).

Akihiko Ozaki: writing (review and editing).

Aya Goto: conceptualisation, methodology, project administration, supervision, writing (review and editing).

## ETHICS STATEMENT

This paper reports analysis of primary data. The ethics of data collection and analysis were approved by the Research Ethics Committee of the School of Social and Political Science, University of Edinburgh.

## Data Availability

Research data are not shared.^2^

## Endnotes

^1^
For reflections on fully‐informed consent in this context, see also Midorikawa and Ohtsuru (2020).

## References

[disa12681-bib-0001] Abeysinghe, S. and C. Leppold (2023) ‘Ethical research practice in health and disasters’. International Journal of Disaster Risk Reduction. 92 (June). Article number: 103728. 10.1016/j.ijdrr.2023.103728.

[disa12681-bib-0002] Adams, R.M. , C.M. Evans , and L. Peek (2024) ‘Defining, collecting, and sharing perishable disaster data’. Disasters. 48(1). Article number: e12592. 10.1111/disa.12592. 37212533

[disa12681-bib-0003] Adams‐Hutcheson, G. (2017) ‘Mobilising research ethics: two examples from Aotearoa New Zealand’. New Zealand Geographer. 73(2). pp. 87–96.

[disa12681-bib-0004] Allden, K. et al. (2009) ‘Mental health and psychosocial support in crisis and conflict: report of the Mental Health Working Group’. Prehospital and Disaster Medicine. 24(S2). pp. S217–S227. 19806544 10.1017/s1049023x00021622

[disa12681-bib-0005] Bovaird, T. , G.G. Van Ryzin , E. Loeffler , and S. Parrado (2015) ‘Activating citizens to participate in collective co‐production of public services’. Journal of Social Policy. 44(1). pp. 1–23.

[disa12681-bib-0006] Braun, V. and V. Clarke (2006) ‘Using thematic analysis in psychology’. Qualitative Research in Psychology. 3(2). pp. 77–101.

[disa12681-bib-0007] Brun, C. (2009) ‘A geographers’ imperative? Research and action in the aftermath of disaster’. The Geographical Journal. 175(3). pp. 196–207.

[disa12681-bib-0008] Chiumento, A. , A. Rahman , L. Frith , L. Snider , and W.A. Tol (2017) ‘Ethical standards for mental health and psychosocial support research in emergencies: review of literature and current debates’. Globalization and Health. 13 (February). Article number: 8. 10.1186/s12992-017-0231-y. 28178981 PMC5299703

[disa12681-bib-0009] Collogan, L.K. , F. Tuma , R. Dolan‐Sewell , S. Borja , and A.R. Fleischman (2004) ‘Ethical issues pertaining to research in the aftermath of disaster’. Journal of Traumatic Stress. 17(5). pp. 363–372. 15633915 10.1023/B:JOTS.0000048949.43570.6a

[disa12681-bib-0010] Elliott, D. (2013) Fukushima: Impacts and Implications. Palgrave Macmillan, Basingstoke.

[disa12681-bib-0011] Filipe, A. , A. Renedo , and C. Marston (2017) ‘The co‐production of what? Knowledge, values, and social relations in health care’. PLoS Biology. 15(5). Article number: e2001403. 10.1371/journal.pbio.2001403. 28467412 PMC5414996

[disa12681-bib-0012] Funtowicz, S.O. and J.R. Ravetz (1993) ‘Science for the post‐normal age’. Futures. 25(7). pp. 739–755.

[disa12681-bib-0013] Gaillard, J‐C. and L. Peek (2019) ‘Disaster‐zone research needs a code of conduct’. Nature. 575(7783). pp. 440–442. 31748720 10.1038/d41586-019-03534-z

[disa12681-bib-0014] Galende‐Sánchez, E. and A.H. Sorman (2021) ‘From consultation toward co‐production in science and policy: a critical systematic review of participatory climate and energy initiatives’. Energy Research & Social Science. 73 (March). Article number: 101907. 10.1016/j.erss.2020.101907.

[disa12681-bib-0015] Gibbs, L. et al. (2018) ‘Distress and satisfaction with research participation: impact on retention in longitudinal disaster research’. International Journal of Disaster Risk Reduction. 27(March). pp. 68–74.

[disa12681-bib-0016] Howard, J. (2011) ‘Environmental nasty surprise, post‐normal science, and the troubled role of experts in sustainable democratic environmental decision making’. Futures. 43(2). pp. 182–195.

[disa12681-bib-0017] Howlett, M. , A. Kekez , and O‐O. Poocharoen (2017) ‘Understanding co‐production as a policy tool: integrating new public governance and comparative policy theory’. Journal of Comparative Policy Analysis: Research and Practice. 19(5). pp. 487–501.

[disa12681-bib-0018] Ingham, V. , M.R. Islam , J. Hicks , A. Lukasiewicz , and C. Kim (2022) ‘Definition and explanation of community disaster fatigue’. In A. Lukasiewicz and T. O'Donnell (eds.) Complex Disasters: Compounding, Cascading, and Protracted. Palgrave Macmillan, Singapore. pp. 341–361.

[disa12681-bib-0019] Kimura, A.H. (2019) ‘Citizen science in post‐Fukushima Japan: the gendered scientization of radiation measurement’. Science as Culture. 28(3). pp. 327–350.

[disa12681-bib-0020] Kita, S.M. (2017) ‘Researching peers and disaster vulnerable communities: an insider perspective’. The Qualitative Report. 22(10). pp. 2600–2611.

[disa12681-bib-0021] Kobashi, Y. , T. Sawano , A. Crump , M. Kami , and M. Tsubokura (2020) ‘Unambiguous evidence is required to accurately understand the health impacts of nuclear accidents’. Journal of Radiation Research. 61(1). pp. 90–91. 31740952 10.1093/jrr/rrz069 PMC6976740

[disa12681-bib-0022] Koyama, Y. (2022) ‘Living with contradiction – risk perception, self‐determination, and life after Fukushima’. Environmental Advances. 7 (April). Article number: 100181. 10.1016/j.envadv.2022.100181.

[disa12681-bib-0023] Lambert, N. and S. Carr (2018) ‘“Outside the original remit”: co‐production in UK mental health research, lessons from the field’. International Journal of Mental Health Nursing. 27(4). pp. 1273–1281. 29920906 10.1111/inm.12499

[disa12681-bib-0024] Lesen, A.E. , C. Tucker , M.G. Olson , and R.J. Ferreira (2019) ‘“Come back at us”: reflections on researcher‐community partnerships during a post‐oil spill Gulf Coast resilience study’. Social Sciences. 8(1). Article number: 8. 10.3390/socsci8010008.

[disa12681-bib-0025] Levine, C. (2004) ‘The concept of vulnerability in disaster research’. Journal of Traumatic Stress. 17(5). pp. 395–402. 15633918 10.1023/B:JOTS.0000048952.81894.f3

[disa12681-bib-0026] Louis‐Charles, H.M. , R. Howard , L. Remy , F. Nibbs , and G. Turner (2020) ‘Ethical considerations for postdisaster fieldwork and data collection in the Caribbean’. American Behavioral Scientist. 64(8). pp. 1129–1144.

[disa12681-bib-0027] Matsumoto, M. (2013) ‘“Structural disaster” long before Fukushima: a hidden accident’. Development and Society. 42(2). pp. 165–190.

[disa12681-bib-0028] Mezinska, S. , P. Kakuk , G. Mijaljica , M. Waligóra , and D.P. O'Mathúna (2016) ‘Research in disaster settings: a systematic qualitative review of ethical guidelines’. BMC Medical Ethics. 17 (October). Article number: 62. 10.1186/s12910-016-0148-7. 27769232 PMC5073437

[disa12681-bib-0029] Midorikawa, S. and A. Ohtsuru (2020) ‘Disaster‐zone research: make participation voluntary’. Nature. 579(7798). Article number: 193. 10.1038/d41586-020-00695-0. 32157232

[disa12681-bib-0030] Miyazawa, K. (2018) ‘Becoming an insider and an outsider in post‐disaster Fukushima’. Harvard Educational Review. 88(3). pp. 334–354.

[disa12681-bib-0031] Nowotny, H. , P.B. Scott , and M.T. Gibbons (2001) Re‐Thinking Science: Knowledge and the Public in an Age of Uncertainty. Polity Press, Cambridge.

[disa12681-bib-0032] O'Mathúna, D.P. (2010) ‘Conducting research in the aftermath of disasters: ethical considerations’. Journal of Evidence‐Based Medicine. 3(2). pp. 65–75. 21349047 10.1111/j.1756-5391.2010.01076.x

[disa12681-bib-0033] Olesen, B.R. and H.M. Nordentoft (2013) ‘Walking the talk? A micro‐sociological approach to the co‐production of knowledge and power in action research’. International Journal of Action Research. 9(1). pp. 67–94.

[disa12681-bib-0034] Oulahen, G. , B. Vogel , and C. Gouett‐Hanna (2020) ‘Quick response disaster research: opportunities and challenges for a new funding program’. International Journal of Disaster Risk Science. 11(October). pp. 568–577.

[disa12681-bib-0035] Patel, S.S. , R.K. Webster , N. Greenberg , D. Weston , and S.K. Brooks (2020) ‘Research fatigue in COVID‐19 pandemic and post‐disaster research: causes, consequences and recommendations’. Disaster Prevention and Management. 29(4). pp. 445–455. 33679011 10.1108/DPM-05-2020-0164 PMC7932124

[disa12681-bib-0036] Polleri, M. (2019) ‘Conflictual collaboration: citizen science and the governance of radioactive contamination after the Fukushima nuclear disaster’. American Ethnologist. 46(2). pp. 214–226.

[disa12681-bib-0037] Polleri, M. (2020) ‘Post‐political uncertainties: governing nuclear controversies in post‐Fukushima Japan’. Social Studies of Science. 50(4). pp. 567–588. 31749407 10.1177/0306312719889405

[disa12681-bib-0038] Pyles, L. (2015) ‘Participation and other ethical considerations in participatory action research in post‐earthquake rural Haiti’. International Social Work. 58(5). pp. 628–645.

[disa12681-bib-0039] Ravetz, J. (2004) ‘The post‐normal science of precaution’. Futures. 36(3). pp. 347–357.

[disa12681-bib-0040] Refsdal, N.O. (2014) ‘Experiences from coordinating research after the 2011 terrorist attacks in Norway’. European Journal of Psychotraumatology. 5(1). Article number: 23215. 10.3402/ejpt.v5.23215. PMC4082193 25018857

[disa12681-bib-0041] Saloranta, T.M. (2001) ‘Post‐normal science and the global climate change issue’. Climatic Change. 50(September). pp. 395–404.

[disa12681-bib-0042] Sawano, T. et al. (2023) ‘The responsibility of the Japanese media, the Fukushima accident and the use of personal data for research’. QJM: An International Journal of Medicine. 116(8). pp. 625–627. 31350887 10.1093/qjmed/hcz193 PMC10497179

[disa12681-bib-0043] Schobert, M. et al. (2023) ‘How do we know vulnerability when we see it? An approach to integrating ethical reflections into empirical disaster research’. International Journal of Disaster Risk Reduction. 97 (October). Article number: 104049. 10.1016/j.ijdrr.2023.104049.

[disa12681-bib-0044] Science Council of Japan (2013) *Academic Research Related to the Great East Japan Earthquake: About Issues and the Future* (in Japanese). March. https://www.scj.go.jp/ja/info/kohyo/pdf/kohyo-22-t170-1.pdf (last accessed on 17 February 2025).

[disa12681-bib-0045] Smith, H. et al. (2022) ‘Co‐production practice and future research priorities in United Kingdom‐funded applied health research: a scoping review’. Health Research Policy and Systems. 20 (April). Article number: 36. 10.1186/s12961-022-00838-x. 35366898 PMC8976994

[disa12681-bib-0046] Tansey, C.M. et al. (2017) ‘Familiar ethical issues amplified: how members of research ethics committees describe ethical distinctions between disaster and non‐disaster research’. BMC Medical Ethics. 18 (June). Article number: 44. 10.1186/s12910-017-0203-z. 28659166 PMC5490228

[disa12681-bib-0047] Thomas‐Hughes, H. (2018) ‘Ethical “mess” in co‐produced research: reflections from a UK‐based case study’. International Journal of Social Research Methodology. 21(2). pp. 231–242.

[disa12681-bib-0048] Van Brown, B.L. (2020) ‘Disaster research “methics”: ethical and methodological considerations of researching disaster‐affected populations’. American Behavioral Scientist. 64(8). pp. 1050–1065.

[disa12681-bib-0049] Wordsworth, R. , C.M. Hall , G. Prayag , and S. Malinen (2021) ‘Critical perspectives on disaster and crisis research: revealing and responding to vulnerability’. In A.D. Hill et al. (eds.) Research in Times of Crisis: Research Methods in the Time of COVID‐19. Volume 13. Emerald Publishing Limited, Bingley. pp. 75–97.

[disa12681-bib-0050] Wyborn, C. et al. (2019) ‘Co‐producing sustainability: reordering the governance of science, policy, and practice’. Annual Review of Environment and Resources. 44(1). pp. 319–346.

[disa12681-bib-0051] Yamamoto, T et al. (2015) ‘Investigative research projects related to the Tohoku earthquake (the Great East Japan Earthquake) conducted in Fukushima’. Fukushima Journal of Medical Science. 61(2). pp. 155–159. 26632193 10.5387/fms.2015-22 PMC5131591

[disa12681-bib-0052] Yasui, S. (2013) ‘An analysis of the argument over the health effects of low‐dose radiation exposure caused by the accident at the Fukushima Daiichi APP in Japan’. Journal of Risk Research. 16(8). pp. 937–944.

